# The effect of posture on airflow distribution, airway geometry and air velocity in healthy subjects

**DOI:** 10.1186/s12890-022-02276-5

**Published:** 2022-12-15

**Authors:** Kris M. Ides, Wilfried A. De Backer, Maarten Lanclus, Glenn Leemans, Wendel Dierckx, Eline Lauwers, Dirk Vissers, Jan Steckel, Jan W. De Backer

**Affiliations:** 1 FLUIDDA NV, Groeningenlei 132, 2550 Kontich, Belgium; 2 Department of Pediatric Medicine, Antwerp University Hospital, drie eikenstraat 655, 2650 Edegem, Belgium; 3 Laboratory of Experimental Medicine and Pediatrics, University of Antwerp, Universiteitsplein 1, 2610 Wilrijk, Belgium; 4 Cosys-Lab, Antwerp University, Flanders Make Lommel, Groenenborgerlaan 171, 2020 Antwerp, Belgium; 5 Faculty of Medicine and Health Sciences, University of Antwerp, Universiteitsplein 1, 2610 Wilrijk, Belgium; 6 FLUIDDA Inc, 228 EAST 45TH Street STE 9E, New York, USA; 7 Medimprove Multidisciplinairy Private Practice, Groeningenlei 132C, 2550 Kontich, Belgium

**Keywords:** Posture, Ventilation, Internal airflow distribution, Respiratory physiology, Airway clearance

## Abstract

**Background:**

Gravity, and thus body position, can affect the regional distribution of lung ventilation and blood flow. Therefore, body positioning is a potential tool to improve regional ventilation, thereby possibly enhancing the effect of respiratory physiotherapy interventions. In this proof-of-concept study, functional respiratory imaging (FRI) was used to objectively assess effects of body position on regional airflow distribution in the lungs.

**Methods:**

Five healthy volunteers were recruited. The participants were asked during FRI first to lie in supine position, afterwards in standardized right lateral position.

**Results:**

In right lateral position there was significantly more regional ventilation also described as Imaging Airflow Distribution in the right lung than in the left lung (*P* < 0.001). Air velocity was significantly higher in the left lung (*P* < 0.05). In right lateral position there was significantly more airflow distribution in the right lung than in the left lung (*P* < 0.001). Significant changes were observed in airway geometry resulting in a decrease in imaged airway volume (*P* = 0.024) and a higher imaged airway resistance (*P* = 0.029) in the dependent lung. In general, the effect of right lateral position caused a significant increase in regional ventilation (*P* < 0.001) in the dependent lung when compared with the supine position.

**Conclusions:**

Changing body position leads to significant changes in regional lung ventilation, objectively assessed by FRI The volume based on the imaging parameters in the dependent lung is smaller in the lateral position than in the supine position. In right lateral decubitus position, airflow distribution is greater in dependent lung compared to the nondependent lung.

*Trial registration*: The trial has been submitted to www.clinicaltrials.gov with identification number NCT01893697 on 07/02/2013.

**Supplementary Information:**

The online version contains supplementary material available at 10.1186/s12890-022-02276-5.

## Background

Distribution of ventilation, or regional ventilation, has long been of interest to clinicians working with patients with a range of pulmonary diseases. In respiratory physiotherapy, improvement of regional ventilation is one of the treatment goals in the management of those patients [1, 2]. Many techniques are described which aim to enhance the regional distribution of ventilation, whereas body positioning is one of them [3]. It is widely accepted that changes in posture produce different spatial patterns of ventilation distribution within the lung [4, 5]. The changes in regional ventilation induced by postural positions are used in many airway clearance techniques to facilitate mucus clearance [6–9]. The underlying mechanism behind mucus clearance in patients with mucociliary dysfunction is the exertion of shearing forces onto sputum at the air–liquid interface. This is described as two-phase gas liquid flow mechanism [10]. Airflow velocity plays a crucial role in the transportation of mucus and could be manipulated by changes in airflow, lung volumes, pulmonary pressures and airway diameter [10, 11]. To accomplish such an optimal mucus transport, airflow velocity can be increased by narrowing the airway diameter in combination with a local increase of airflow [12]. Unfortunately, this physiological principle of airflow velocity is based on models, often not evaluated in human subjects [13]. Recently, the influence of posture has been evaluated in the lateral decubitus position and resulted in a greater clearance of mucus [14, 15]. Martin and colleague’s suggested that this may be due to a better deflation of the dependent lung. Nevertheless, it remains unclear if this greater clearance of mucus was the effect of regional changes in ventilation, airway geometry and air velocity in the dependent lung. These regional changes in ventilation cannot be measured by means of conventional outcome measurements, for example classic lung function tests [14]. However, Functional respiratory imaging (FRI) is able to cater this need by calculating regional changes such as airway diameter, volume and resistance in patient-specific airway models. FRI starts with the acquisition of low dose, high-resolution computed tomography (HRCT) scans of the patient. Measurements are performed on the segmented 3-dimensional geometries from these scans. In a final step Computational Fluid Dynamics (CFD) is used to quantify airflow and its related parameters. In addition, repetitive FRI measures, based on scans at functional residual capacity (FRC) and total lung capacity (TLC) levels, are able to assess lobar expansion, which is an indirect measure of airflow or regional distribution in a specific part of the lung [16]. The main aim of this study is to investigate the effects of posture on flow distribution within the lungs.

## Methods

To unravel the relation between posture and air velocity, we used FRI in this clinical trial to determine if the regional changes in a dependent lung in a lateral position leads to a higher air velocity in comparison with the other lung. Preliminary data were presented in abstract form [17]. The study was approved by the local ethical committee (UZA 13/4/42) and a Trail registration was made: The trial has been submitted to www.clinicaltrials.gov with identification number NCT01893697 on 07/02/2013.

### Study design and subjects

Healthy subjects who volunteered to participate in this observational cross-sectional study were recruited by the research team from relatives or students at the University of Antwerp. Students were invited by e-mail with information about the clinical trial. Inclusion ran from April 2013 to September 2014. Participants were eligible for inclusion if they met following criteria: (1) male or female volunteer; (2) age > 18 years; (3) no respiratory disease in two weeks prior to enrollment; (4) able to perform lung function tests. Participants were excluded if they had (1) an acute or chronic disease; (2) deformities or complications preventing patients to maintain side lying position during the scanning procedure; (3) one or multiple CT scans of the chest during the last year. All participants were screened by research personnel with whom the subjects were not familiar.

### Screening

Demographics, medical history and current use of medication or other non-pharmacological therapies were recorded after obtaining informed consent. The respiratory physician performed a physical examination and verified medical history and ongoing pharmacological and non-pharmacological therapies to ensure good health. Lung function was performed in the lung function laboratory according to ERS standards [18] using Jaeger 5.1 devices. All tests were repeated, until three technically acceptable measurements were made [19]. The lung function tests yielded the following parameters: forced volume in 1 second (FEV1), Tiffenau index (FEV1/ FVC) and peak expiratory flow (PEF) from the spirometry; and airway resistance (Raw), specific airway resistance (sRaw), FRC and TLC from body plethysmography. If the FEV1 or functional vital capacity (FVC) was < 80% of the predicted value, patients were excluded from the study and referred to a respiratory physician for further medical screening.

#### Interventions

In order to investigate the effect of a lateral posture on airflow distribution, airway geometry and air velocity, the body position of the heathy participants was changed in a well-defined order. The participants were asked during functional respiratory imaging first to lie in a supine position (SP), afterwards in a standardized right lateral position (RLP). The right lateral position was used to examine the effects of side lying in the different lobes. The right position includes the effects of gravity on the 3 right lobes. And more over on the reaction of the right middle lobe.

### Procedures

#### Functional respiratory imaging

Functional respiratory imaging (FRI) is a clinically validated computational work-flow in which functional data are added to respiratory anatomic images [16]. Starting with low-dose-HRCT scans, 3-dimensional models of airways and lung models are extracted. The low-dose scanner settings protocol results in an estimated radiation dose of ca. 2.5 mSv for each scan. When performing this extraction on HRCT scans that are taken during breath holding at 2 (spirometry-controlled) distinct lung levels FRC and TLC), it is now possible to assess the geometric changes in airways and lung lobes during the breathing cycle. The measurement was preceded by a training session with the breath holds at TLC and FRC level when patients where positioned in the scanner. These data are then used as boundary conditions for CFD simulations, from which functional information such as flow behavior can be simulated. A detailed description of the FRI methodology was provided by De Backer et al. [16].

#### Outcome parameters

##### Lung or lobe volumes

The lung lobes were segmented both at TLC and FRC by identifying the fissure planes and subsequently uses these surfaces as a separators. For each lobe, the volume was measured (iVlobe) at FRC and TLC. Afterwards the volume of each lobe for a specific lung was counted up to calculate the total volume of respectively the right or left lung.

##### Airflow distributions

The total lobar expansion from FRC to TLC was considered a measure for regional ventilation in the right or the left lung, as this represents the internal airflow distribution as defined in the following equation:

$${\text{IAD}}\,{\text{lobe}} \,\left( \% \right)\, = \,{1}00*\left( {{\text{VTLC}}\_{\text{lobe}}\, - \,{\text{VFRC}}\_{\text{lobe}}/{\text{VTLC}}\_{\text{lungs}} \, - \,{\text{VFRC}}\_{\text{lungs}}} \right)$$

in which IAD lobe is the internal airflow distribution to a specific lobe, VTLC_lobe is the volume of that lobe at TLC, VFRC_lobe is the volume of that lobe at FRC, VTLC_lungs is the total volume of all lobes at TLC, and VFRC_lungs is the total volume of all lobes at FRC.

##### Airway geometry

The parameters iVaw and iRaw which represent airway geometry were obtained by FRI, iVaw is the distal airway volume of individual airways or in different regions, starting at the segmental level of each lobe. iRaw represents the changes in airway resistance of individual airways or different regions. Resistance was defined as the total pressure drop of an airway divided by the flow rate trough that airway.

##### Air velocity

This parameter is calculated using CFD method and defined as the average velocity magnitude towards a certain lobe.

##### Sample size

Sample size calculation was not possible, since no existing data was available of the mean and standard deviation of the primary outcome parameters in healthy subjects. We anticipated to enroll 10 healthy subjects. Even though FRI is based on a low-dose-HRCT scans, a preliminary analysis was done on 5 patients to minimize the number of healthy subjects to exposure of radiation.

### Statistical analysis

Statistical analysis was performed using R version 3.0.2 (The R Foundation for Statistical Computing, Vienna, Austria). Significance level was set at 0.05. Differences between side and supine are assessed using a paired t-test. Differences between the left and right lung for a given posture are quantified using a Welch two sample t-test.

## Results

Five healthy young men were enrolled is this observational study. The subjects, whose physical characteristics and lung function are given in Table 1, showed similar physical and functional characteristics. Firstly, the baseline values for each outcome parameter are described for the right and left lung in both the supine as the right lateral position. Afterwards, the effect of the right lateral position is given on airflow distribution, airway geometry and air velocity for the right and left lung.

**Table 1:** Characteristics of the study group

Subjects (n)	5
Age (years)	25.4 ± 5.3
Height (cm)`	177.2 ± 5.3
Weight (kg)	73 ± 8.2
BMI (kg/m^2^)	23.9 ± 2.5
FEV1 (L)	5.1 ± 0.7
FEV1 (% pred)	118.4 ± 13.2
FEV1/FVC (%)	84.1 ± 4.8
VC (L)	6.1 ± 0.8
VC (% pred)	113.8 ± 12.4
FRC (L)	3.7 ± 0.6
FRC (% pred)	112.0 ± 16.7
RV (L)	1.8 ± 0.2
RV (% pred)	105.4 ± 7.8
TLC (L)	7.9 ± 0.8
TLC (% pred)	112.0 ± 11.0
Raw (kPa.s/L)	0.2 ± 0.1
sRaw (kPa.s)	0.9 ± 0.3

Data are presented as mean ± SD

BMI, body mass index; FEV1, forced expiratory volume in one second; FVC, forced vital capacity; VC, vital capacity; FRC, functional residual capacity; RV, residual volume; TLC, total lung capacity; iRaw, airway resistance; siRaw, specific airway resistance; % pred, % predicted

### Supine position (SP): left versus right lung

In supine position, regional ventilation (IAD) was significantly greater in the right lung than in the left lung (*P* < 0.001). The air velocity was significantly higher in the left lung (*P* < 0.05). There were no significant differences for the other outcome parameters. For a detailed view of all outcome parameters, see Table 2.

**Table 2:** Baseline values and difference between left and right lung in supine and right lateral position (RLP) by functional respiratory imaging (FRI)

	Left lung		Right lung		△Left versus right lung	p-value
	Mean	SD	Mean	SD		
*Supine position*						
Vlobes FRC (L)	1.819	0.445	2.127	0.425	right > left	0.296
Vlobes TLC (L)	3.529	0.450	3.967	0.485	right > left	0.177
IAD (%)	48.158	0.951	51.842	0.951	right > left	< 0.001
iVaw (mL)	8.804	1.404	9.089	1.868	right > left	0.311
iRaw (kPas/L)	0.041	0.012	0.027	0.015	left > right	0.141
Air velocity (m/s)	1.526	0.071	1.196	0.242	left > right	0.035*
*Right lateral position*						
Vlobes FRC (L)	2.604	0.330	1.740	0.395	left > right	0.006*
Vlobes TLC (L)	3.669	0.400	3.649	0.665	left = right	0.890
IAD (%)	34.5	5.18	65.5	5.81	right > left	< 0.001
iVaw (mL)	8.619	1.782	9.887	1.924	right > left	0.793
iRaw (kPas/L)	0.042	0.020	0.037	0.017	right > left	0.693
Air velocity (m/s)	1.235	0.097	1.422	0.272	right > left	0.209

Data are presented as mean ± SD

Vlobe FRC, volume of the lobes at functional residual capacity; Vlobes TLC, volume of the lobes at total lung capacity; IAD, internal airflow distribution; iVaw, distal airway volume; iRaw, airway resistance

*p < *0.05*

### Right lateral position (RLP): left versus right lung

In RLP, regional ventilation (IAD) was significantly greater in the right lung than in the left lung (*P* < 0.001). The volume for the left lung was significantly higher at FRC (*P* < 0.05). There were no significant differences for the other outcome parameters. For a detailed view of all outcome parameters, see Table 2.

### Effect of RLP: supine versus side for the dependent lung

**Fig. 1:**
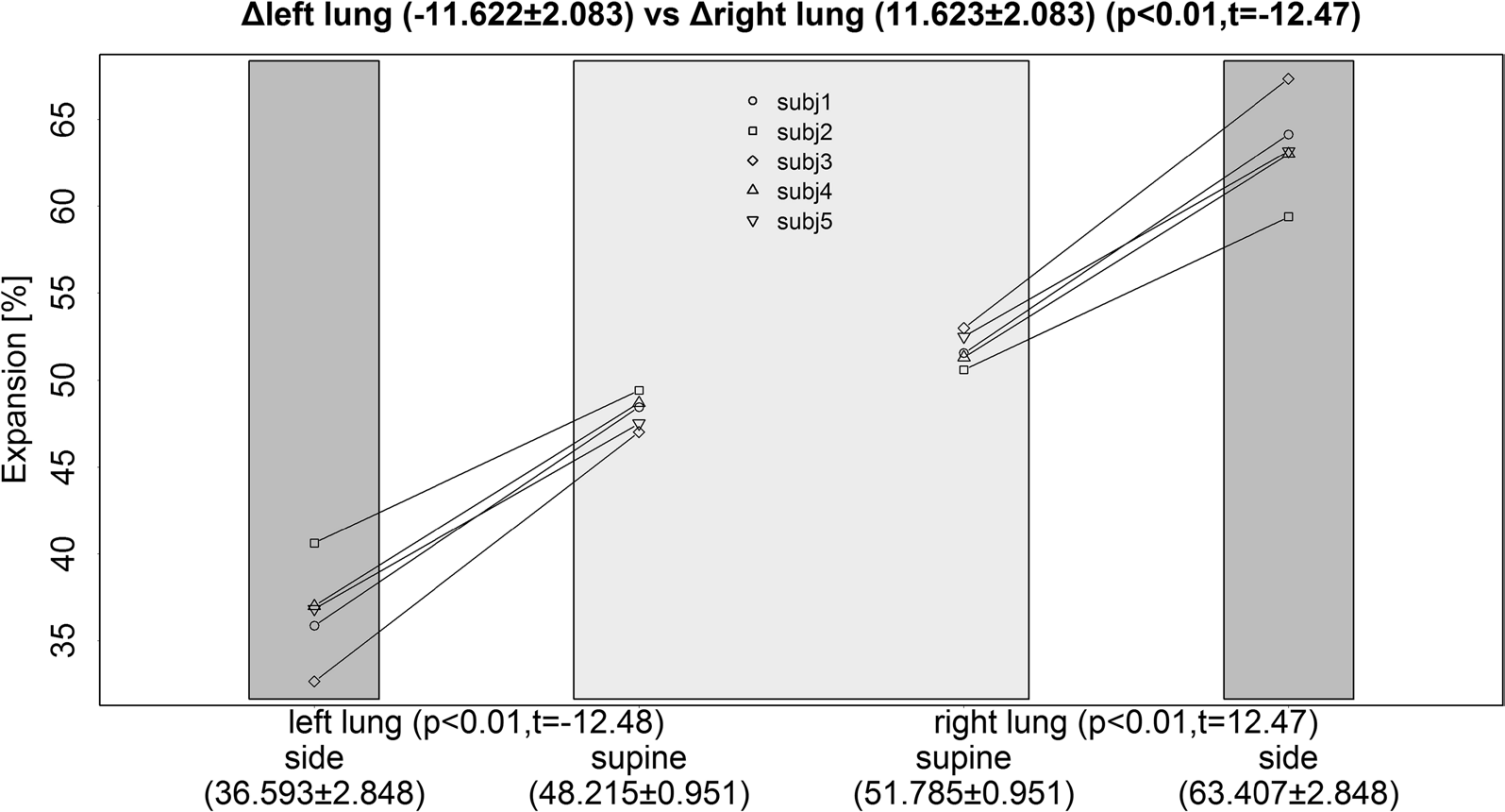
Effect of the right lateral position on regional ventilation (IAD) compared with a supine position for the right and left lung

**Fig. 2:**
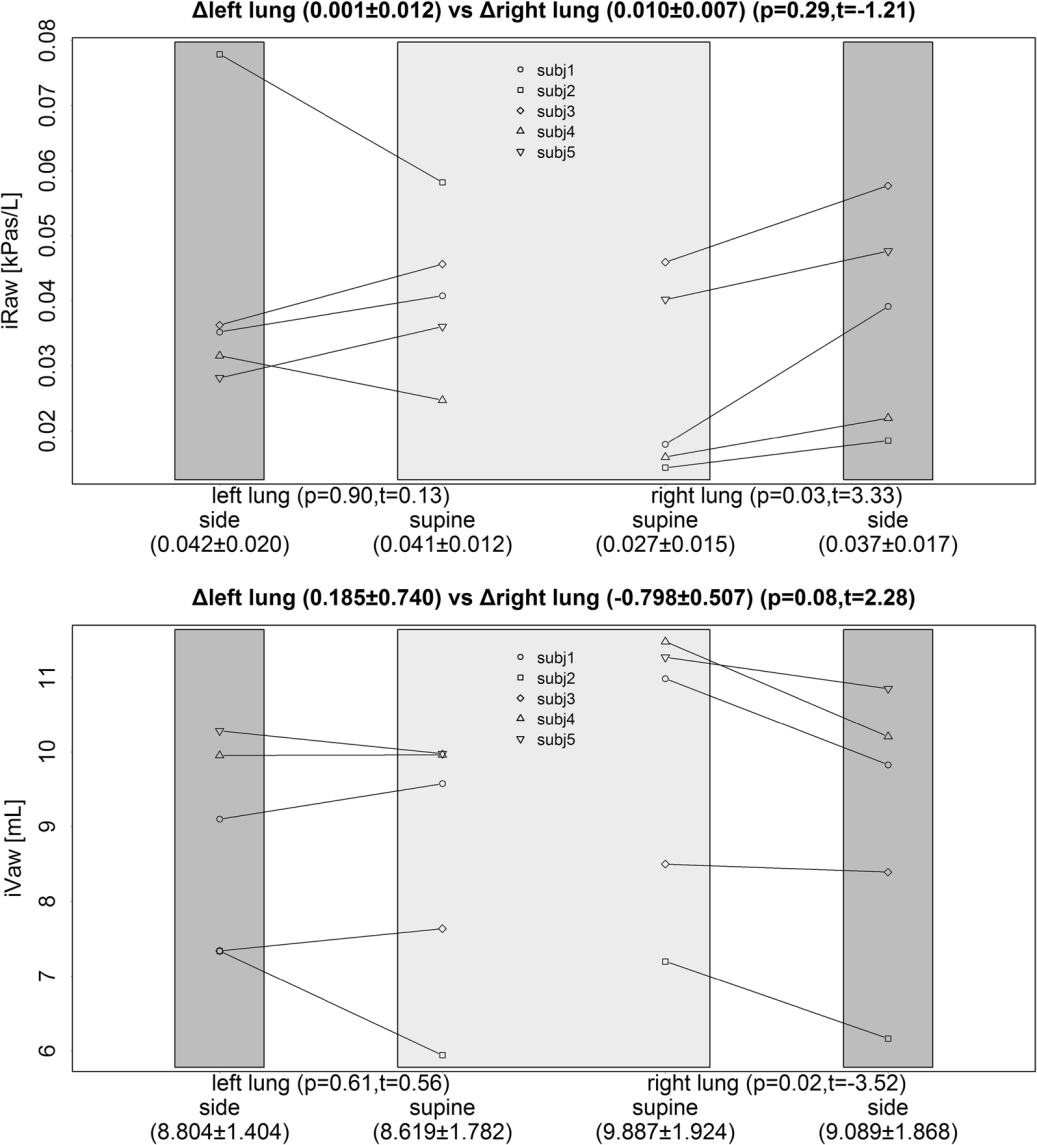
Effect of the right lateral position on airway geometry (iRaw–iVaw) compared with a supine position for the right and left lung

**Fig. 3:**
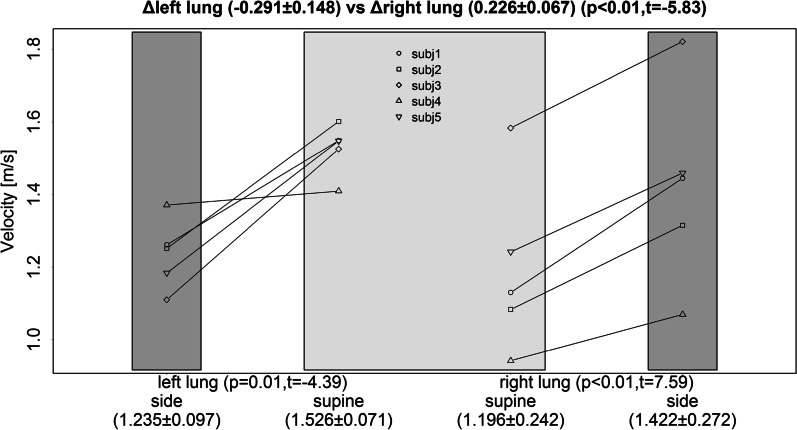
Effect of the right lateral position on air velocity compared with a supine position for the right and left lung

Firstly, the effect of the right lateral position (RLP) causes in the right or dependent lung a significant increase in regional ventilation (IAD; *P* < 0.001) when compared with the supine position. In the left lung, the opposite significant change is observed (Fig. 1). Secondly, significant changes are observed in airway geometry by a decrease in iVaw (*P* = 0.024) and a higher iRaw (*P* = 0.029) in the dependent lung (Fig. 2). On the one hand, the combination of greater regional ventilation and smaller airway geometry results in a significant increase in air velocity in the dependent lung (*P* = 0.002). On the other hand, the decreased regional ventilation, and the unchanged airway geometry in the left lung results in a significant decrease in air velocity (*P* = 0.012) when compared with the supine position (Fig. 3).

**Fig. 4:**
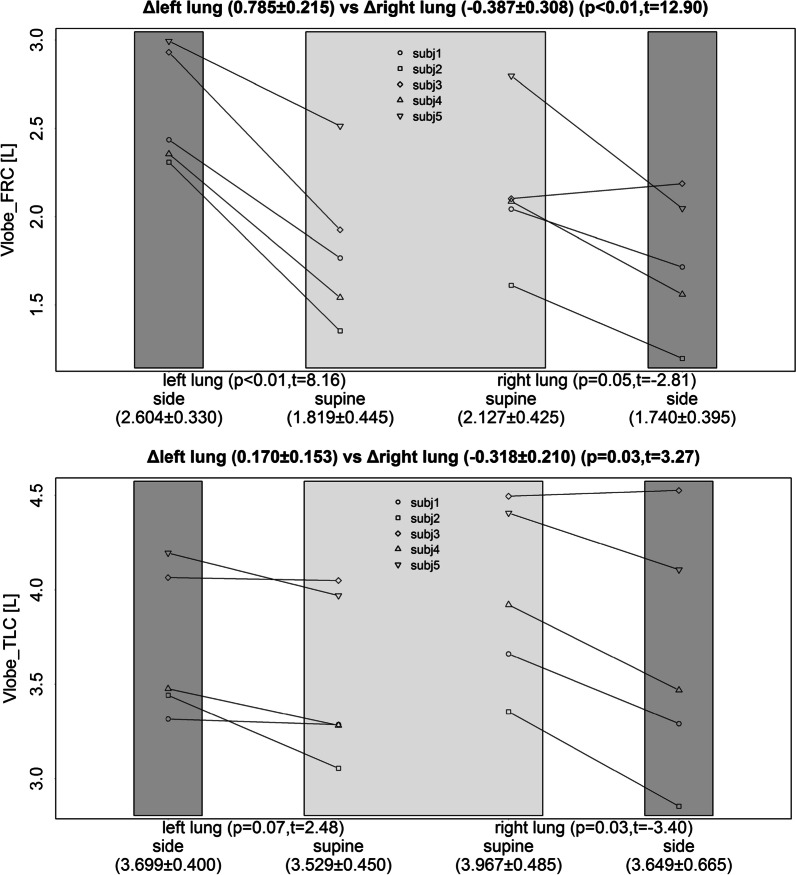
Effect of the right lateral position on Lobar volumes (Vlobe) on Functional residual capacity (FRC) and Total lung capacity (TLC) compared with a supine position for the right and left lung

**Fig. 5:**
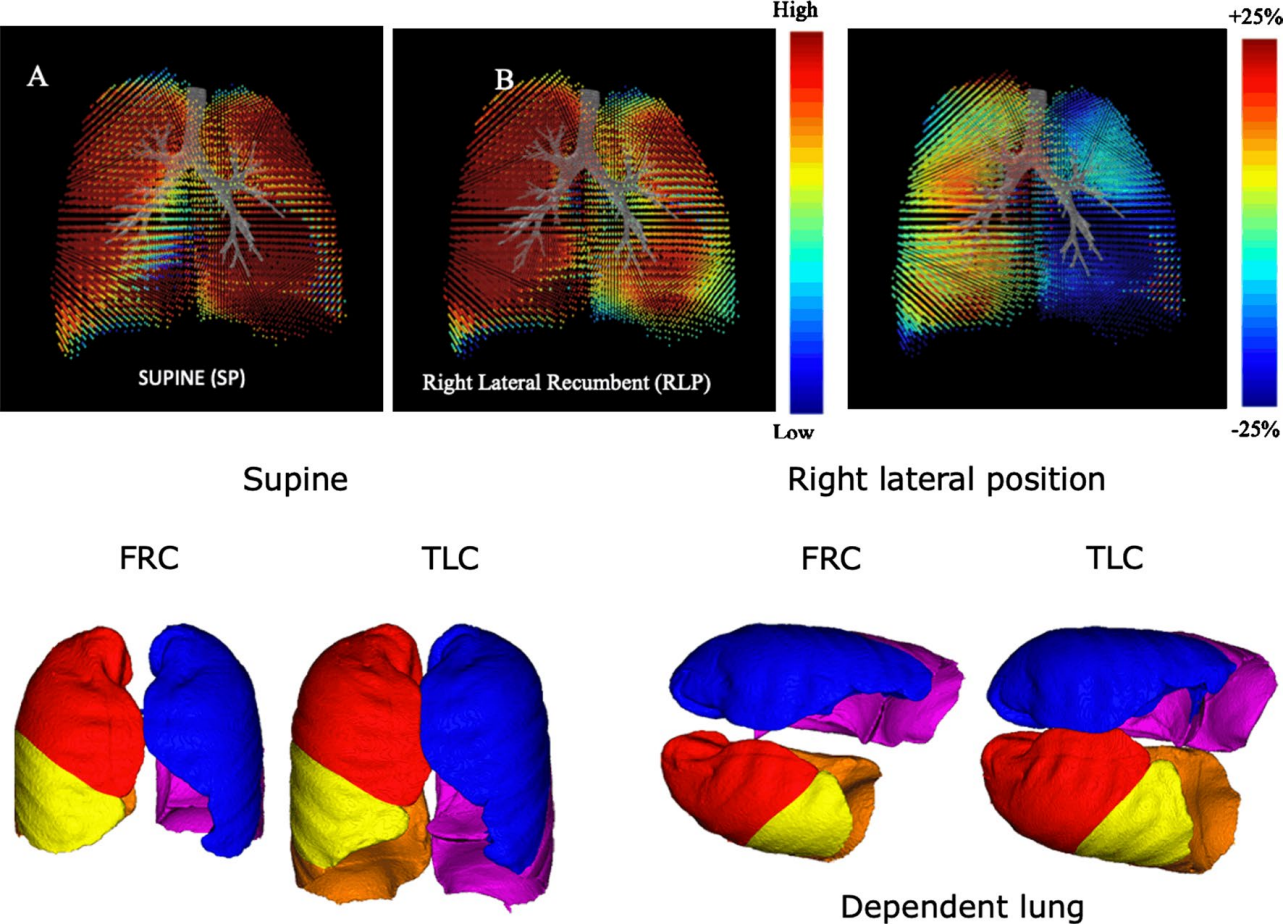
Regional ventilation maps for a healthy subject in supine (**A**) and right lateral position (**B**), as well as the relative effect between both. substantial relative ventilation defect can be seen in the left lung as a result of the right lateral position, with regional effect values up to 25. Ventilation maps are CT based representations of regional ventilation, derived from pulmonary CT images acquired at two different inflation levels, functional residual capacity (FRC) and total lung capacity (TLC) without the use of exogenous contrast, by assuming that regional changes in lung volume relate to regional ventilation The Jacobian determinant of the deformation field from FRC to TLC is calculated, which measures the differential expansion of the image

The lobar volumes in left and right lung where measured and changes are represented in Fig. 4. It is noticeable that the change between tTLC and FRC is apparent. In RLP, a larger deflation of the dependent lung in noticeable. The left lung in RLP receives relatively less air as it is in a stretched position. On the level of TLC, the differences between supine and RLP are les apparent as in the FRC situation. These regional ventilation differences are mapped for a healthy subject in supine and RLP. The relative effect between both RLP and SP are presented in Fig. 5. These ventilation maps show a ventilation decrease in the left lung as a result of the RLP, with regional effect values up to 25%. Ventilation maps are CT based representations of regional ventilation, derived from pulmonary CT images acquired at two different inflation levels, FRC and TLC (without the use of exogenous contrast), by assuming that regional changes in lung volume relate to regional ventilation. The Jacobian determinant of the deformation field from FRC to TLC is calculated, which measures the differential expansion of the image (Additional file 1).

## Discussion

This paper investigates the effects of posture on lung ventilation, to unravel the relation between lateral positioning and the facilitation of mucus clearance. This mucus clearance is well used in respiratory physical therapy treatments and focusses on air velocity as driving parameter for mucus displacement. These physical therapy regimes are mainly based on the development of an optimal expiratory airflow velocity that applies shearing forces on the mucus at the inner surface of the airway. On the one hand, gravity plays a major role in the variability of ventilation. Since the lung deforms under its own weight, the intrapleural pressure is less negative at the dependent regions than in the upper regions of the lung. This results in changes of static lung volumes like FRC, which are smaller in the upper regions irrespective of the patient’s position [4]. In our paper we could demonstrate similar results using FRI techniques. These changes will result in the dependent regions receiving a greater proportion of the inspired volume at volumes greater than FRC and results in greater regional ventilation. Following this observation, we calculated the airway velocity in the different positions. We could demonstrate a change in airway velocity in favor of the dependent lung, whereas in supine position no differences could be noticed between right and left lung. A higher velocity could result in an increase of shearing forces. These shearing forces eventually lead to displacement of mucus to the central airways were it could be evacuated [8, 20]. To accomplish optimal velocity with the same expiratory airflow, it is necessary to decrease the airway diameter and hence increase the airflow velocity. In other words, the airway diameter and resistance are important factors to consider in mucus clearance techniques. Looking at the images we were able to measure the difference in airway diameter. Results support that the change in airway velocity is caused by the decrease in airway diameter for the dependent lung. For the parent lung no changes in diameter could be observed. Alcoforado et al. suggested how pulmonary ventilation behaves in the lateral position [20, 21]. Our results support these findings showing greater regional ventilation in the dependent lung. Although we looked at healthy volunteers our findings can support these of Martins et al. They found a significant increase in airway clearance in the peripheral airways of the dependent lung in patients with chronic bronchitis [14]. This higher clearance rate of the dependent lung can be facilitated by a gentle narrowing of the airway diameters by 4% of the peripheral airways resulting in a higher flow velocity and optimal use of shearing forces. Dean et al. described the benefits of lateral position on the dependent lung whit special interest for improving PaO_2_ [22]. The lateral position places the inferior portion diaphragm in a mechanically advantage position resulting in a greater excursion. Functional residual capacity is known to be reduced in lateral position. Airway closure and alveolar hypoventilation are apparently potentiated in the dependent lung. They also found that ventilation is forced to non-dependent areas and perfusion towards dependent areas [22]. The results of the study performed by Alcoforado et al. suggest that quantitatively as qualitatively, aerosol deposition during nebulization is induced in the dependent areas of lungs when using a diaphragmatic respiratory pattern associated with a lateral decubitus position [23]. Our findings suggest less deflation in the parent lung. On the other hand, the dependent lung shows a greater % in volume expansion. The lung starts from a smaller volume and does not reach the same volume as seen in the supine position. Local flow distribution however shows a greater flow towards the dependent lung [24]. Presumed effectiveness of positioning patients and its application may be based more on tradition and anecdotal report than on scientific evidence. The procedure has been used excessively and in patients in whom it is not indicated [25]. We now have data to support the underlying hypothesis for the change of regional redistribution, airway resistance and change in airway diameter and lobar volume. The results could be of further importance for further use in treating respiratory diseases.

### Limitations

In this proof-of-concept study we focused on male participants only, to exclude possible gender differences. It is documented in literature that gender related difference exist in airway morphology [26], so these results should be interpreted with some caution for female subjects. This is a study on young healthy volunteers. The small number of participants in this study can be seen as a limiting factor. Despite this small number, the study gives an insight in the lung and flow mechanics of healthy people. Extrapolation of these results must be done with caution as it is known that some respiratory diseases might alter lung structure and mechanics. It provides a basis to cautiously support the current hypothesis. These findings support the theoretical hypothesis for the use of side lying positioning as an aid in applying higher shearing forces during airway clearance therapy.

## Conclusion

In summary, FRI has the potential to visualize and report possible changes in airway diameter and airflow distributions due to lateral posture. Changing body position leads to significant changes in regional lung ventilation, objectively assessed by FRI. The volume based on the imaging parameters in the dependent lung is smaller in the lateral position than in the supine position. In right lateral decubitus position, airflow distribution is greater in dependent lung compared to the nondependent lung. In combination with a smaller airway diameter, a right lateral lying position leads to a higher air velocity in the dependent lung. These findings support the theoretical hypothesis for the use of side lying positioning as an aid in applying higher shearing forces during airway clearance therapy. However, this study assessed only a limited number of patients and more research is needed to assess differences in regional ventilation. The physiological effects from different, positions and their possible role in respiratory physiotherapy should be assessed.

## Supplementary Information

**Additional file 1.** All collected raw data are presented in the additional file.

## Data Availability

All data generated or analysed during this study are included in this published article [and its Additional file 1].

## Abbreviations

FRI: Functional respiratory imaging
HRCT: High resolution Computer tomography
CFD: Computational fluid dynamics
TLC: Total lung capacity
FRC: Functional residual capacity
FEV1: Forced expiratory volume in 1 second
FEV1/ FVC: Tiffenau index
PEF: Peak expiratory flow
ERS: European respiratory society
FVC: Functional vital capacity
Raw: Airway resistance
sRaw: Specific airway resistance
SP: Supine position
RLP: Right lateral position
iVLobe: Volume of the lobe from 3D CT image
IAD lobe: Internal airflow distribution form the lobe
iVaw: Distal airway volume of individual airways
iRaw: Represents the changes in airway resistance of individual airways or different regions
iDaw: Distal airway diameter of individual airways
VTLC Lobe: Lobar volume from 3D CT at TLC level
VFRC lobe: Lobar volume from 3D CT at FRC level
VTLC lungs: Lung volume from 3D CT at TLC level
VFRC Lungs: Lung volume from 3D CT at FRC level
PaO_2_: Arterial oxygen pressure

## References

[1] Bott J, Blumenthal S, Buxton M, Ellum S, Falconer C, Garrod R, Harvey A, Hughes T, Lincoln M, Mikelsons C, Potter C, Pryor J, Rimington L, Sinfield F, Thompson C, Vaughn P, White J (2009). Guidelines for the physiotherapy management of the adult, medical, spontaneously breathing patient. Thorax.

[2] Langer D, Hendriks EJM, Burtin C, Probst V, van der Schans CP, Paterson WJ, Verhoef-de Wijk MCE, Straver RVM, Klaassen M, Troosters T, Decramer M, Ninane V, Delguste P, Muris J, Gosselink R (2009). A clinical practice guideline for physiotherapists treating patients with chronic obstructive pulmonary disease based on a systematic review of available evidence. Clin Rehabil.

[3] Galvin I, Drummond GB, Nirmalan M (2007). Distribution of blood flow and ventilation in the lung: gravity is not the only factor. Br J Anaesth.

[4] Kaneko K, Milic-Emili J, Dolovich MB, Dawson A, Bates DV (1966). Regional distribution of ventilation and perfusion as a function of body position. J Appl Physiol.

[5] Musch G, Layfield JDH, Harris RS, Melo MFV, Winkler T, Callahan RJ, Fischman AJ, Venegas JG (2002). Topographical distribution of pulmonary perfusion and ventilation, assessed by PET in supine and prone humans. J Appl Physiol.

[6] Bellone A, Lascioli R, Raschi S, Guzzi L, Adone R (2000). Chest physical therapy in patients with acute exacerbation of chronic bronchitis: effectiveness of three methods. Arch Phys Med Rehabilit.

[7] Varekojis SM, Douce FH, Flucke RL, Filbrun DA, Tice JS, McCoy KS, Castile RG (2003). A comparison of the therapeutic effectiveness of and preference for postural drainage and percussion, intrapulmonary percussive ventilation, and high-frequency chest wall compression in hospitalized cystic fibrosis patients. Respir Care.

[8] Martins J, Andrade A, Assis R, Lara R, Parreira V (2006). The effects of ELTGOL on mucociliary clearance in patients with COPD. Eur Respir Rev.

[9] Pryor JA (1999). Physiotherapy for airway clearance in adults. Eur Respir J.

[10] Kim CS, Rodriguez CR, Eldridge MA, Sackner MA (1986). Criteria for mucus transport in the airways by two-phase gas–liquid flow mechanism. J Appl Physiol.

[11] Van der Schans CP (2007). Bronchial mucus transport. Respir Care.

[12] Van Der Schans CP, Postma DS, Koe GH (1999). Physiotherapy and bronchial mucus transport. Eur Respir J.

[13] Zahm JM, King M, Duvivier C, Pierrot D, Girod S, Puchelle E (1991). Role of simulated repetitive coughing in mucus clearance. Eur Respir J.

[14] Martins JA, Dornelas de Andrade A, Britto RR, Lara R, Parreira VF (2012). Effect of slow expiration with glottis opened in lateral posture (ELTGOL) on mucus clearance in stable patients with chronic bronchitis. Respir Care.

[15] Herrero-Cortina B, Vilaró J, Martí D, Torres A, San Miguel-Pagola M, Alcaraz V, Polverino E (2016). Short-term effects of three slow expiratory airway clearance techniques in patients with bronchiectasis: a randomised crossover trial. Physiotherapy.

[16] De Backer JW, Vos W, Vinchurkar S, Claes R, Drollmann A (2010). Validation of computational fluid dynamics in CT-based. Radiology.

[17] Leemans G, Ides K, Van Holsbeke C, Vissers D, Vos W, De Backer W (2013). The influence of posture on airway structure and function in healthy subjects [abstract]. Eur Respir J.

[18] Frey U, Stocks J, Coates A, Sly P, Bates J (2000). Specifications for equipment used for infant pulmonary function testing. Eur Respir J.

[19] Miller MR, Crapo R, Hankinson J, Brusasco V, Burgos F, Casaburi R, Coates A, Enright P, van der Grinten CPM, Gustafsson P, Jensen R, Johnson DC, MacIntyre N, McKay R, Navajas D, Pedersen OF, Pellegrino R, Viegi G, Wanger J (2005). General considerations for lung function testing. Eur Respir J Off J Eur Soc Clin Respir Physiol.

[20] Dab I, Alexander F (1979). I Dab autogenic drainage studied with flow volume curves.pdf. Monogr Paediatr.

[21] Alcoforado L, Reinaux CMA (2011). Influence of change in lateral decubitus on pulmonary aerosol deposition. Influência da variação dos decúbitos laterais na deposição pulmonar de aerossol.

[22] Dean E (1985). Effect of body position on pulmonary function. Phys Ther.

[23] Alcoforado L, Filho LCP, Brandão DC, Galvão AM, Reinaux CMA, de Andrade AD (2011). Influência Da Variação Dos Decúbitos Laterais Na Deposição Pulmonar De Aerosol. Rev Bras Fisioter.

[24] Washko GR, O’Donnell CR, Loring SH (2006). Volume-related and volume-independent effects of posture on esophageal and transpulmonary pressures in healthy subjects. J Appl Physiol.

[25] Ides K, Vissers D, Vissers D, De Backer L, Leemans G, De Backer W (2011). Airway clearance in COPD: need for a breath of fresh air? A systematic review. COPD.

[26] Kim Y, Schroeder J, Lynch D, Newell J, Make B, Friedlander A, Hanania NA, Washko G, Murphy JR, Wilson C, Hokanson JE, Zach J, Butterfield K, Bowler RP, Investigators CR (2011). Gender differences of airway dimensions in anatomically matched sites on CT in smokers. COPD.

